# Estrogen Receptors and Their Implications in Colorectal Carcinogenesis

**DOI:** 10.3389/fonc.2015.00019

**Published:** 2015-02-02

**Authors:** Francesco Caiazza, Elizabeth J. Ryan, Glen Doherty, Desmond C. Winter, Kieran Sheahan

**Affiliations:** ^1^Centre for Colorectal Disease, Saint Vincent’s University Hospital, Dublin, Ireland; ^2^School of Medicine and Medical Science, University College, Dublin, Ireland; ^3^Department of Surgery, St. Vincent’s University Hospital, Elm Park, Dublin, Ireland; ^4^Department of Pathology, Saint Vincent’s University Hospital, Dublin, Ireland

**Keywords:** estrogen, estrogen receptor, colorectal cancer, tumor immunology, tumor microenvironment

## Abstract

Upon binding their cognate receptors, ERα (ESR1) and ERβ (ESR2), estrogens activate intracellular signaling cascades that have important consequences for cellular behavior. Historically linked to carcinogenesis in reproductive organs, estrogens have also been implicated in the pathogenesis of different cancer types of non-reproductive tissues including the colon. ERβ is the predominant estrogen receptor expressed in both normal and malignant colonic epithelium. However, during colon cancer progression, ERβ expression is lost, suggesting that estrogen signaling may play a role in disease progression. Estrogens may in fact exert an anti-tumor effect through selective activation of pro-apoptotic signaling mediated by ERβ, inhibition of inflammatory signals and modulation of the tumor microenvironment. In this review, we analyze the estrogen pathway as a possible therapeutic avenue in colorectal cancer, we report the most recent experimental evidence to explain the cellular and molecular mechanisms of estrogen-mediated protection against colorectal tumorigenesis, and we discuss future challenges and potential avenues for targeted therapy.

## Introduction

Estrogens (including estrone, estriol, and the biologically active metabolite 17β-estradiol) are cholesterol-derived steroid hormones that are produced by aromatization of androgens primarily in the ovary but also in other tissues (including muscle, adipose, and nervous tissues). Estrogens play a central role in controlling sexual behavior and reproductive functions, and are important in the physiology of both men and women. Estrogens regulate the development and homeostasis of a wide range of tissues and organs, playing a crucial role in the cardiovascular, nervous, and immune systems, as well as in bone metabolism (1). As a result, estrogens have been implicated in numerous pathophysiological conditions including cancer (2). Historically linked to carcinogenesis in reproductive tissues (ovary, uterus, and breast in women; prostate in men), estrogens are also implicated in different cancer types of non-reproductive tissues like the lung and the gastro-intestinal system. The protective role of estrogens against carcinogenesis specifically in the colon was the subject of a previous review (3). Here, we aim to extend our analysis of estrogen signaling in the colon, including the role of the tumor microenvironment and immune evasion mechanisms; we also aim to discuss the potential role of manipulating the estrogen pathway as a therapy in colorectal cancer (CRC).

## Estrogen Receptors: Structure, Function and Mechanisms of Action

Estrogens bind to two different estrogen receptors (ERs), ESR1 (ERalpha, or ERα) and ESR2 (ERbeta or ERβ), both members of the nuclear receptor family (hereafter referred to as ERα and ERβ). Like other members of this family, ERs are modular proteins organized in functional domains (N-terminal domain, DNA-binding domain, hinge region, ligand-binding C-terminal domain) that interact to mediate ligand-dependent gene expression (4, 5). ERα and ERβ, which are encoded by two separate genes (ESR1 and ESR2) located on different chromosomes, share a high degree of sequence homology particularly in the DNA-binding region (97% homology) and to a lesser extent in the ligand-binding region (59% homology), which underlies their shared mechanism of action with differences in their specificities and sensitivities for different ligands. ERα and ERβ also have markedly different tissue- and organ-specific expression patterns (5). In the classical mechanism of action, estrogen-bound ERs located in the cytoplasm undergo conformational changes that allow them to dissociate from scaffolding heat shock proteins (Hsp90 and Hsp70), to dimerize and to translocate to the nucleus, where they can bind to specific DNA sequences (ERE) in the promoter regions of target genes to modulate their expression (4, 6). In addition, ERs can also bind to other transcription factors including Sp1, AP-1, and NFκB, allowing them to extend their influence to genes that do not contain ERE sequences in their promoter regions. Both ERα and ERβ interact with receptor tyrosine kinases, scaffolding proteins, guanine nucleotide exchange (G)-proteins, as well as other intracellular signaling proteins, allowing them to activate a wide range of cytoplasmic signaling pathways, a process, which is independent of transcription but that can ultimately impact on latent gene expression (5, 7, 8). The cumulative action of estrogens (as well as other steroid hormones) ultimately results from the integration of signaling originating from receptors located in different cellular compartments (9). This provides a mechanistic explanation for the delicate fine-tuning of responses to hormonal stimulation that occurs in a tissue-specific fashion and depending on the cellular milieu.

ERα and ERβ show differences in their biological functions dependent on both nuclear and extra-nuclear signaling. Generally, ERα promotes proliferative signaling through differential expression of pro- and anti-apoptotic proteins, as well as cyclin D1 to promote cell cycle transition (10, 11). On the other hand, ERβ acts as a dominant regulator, inducing a reduction in ERα-mediated gene expression (when the two receptors are co-expressed) with a consequent negative effect on cell proliferation (12). ERβ is also able to exert standalone anti-proliferative effects (in the absence of ERα) by activating pro-apoptotic signaling through the p38/MAPK pathway (11, 13).

## Estrogen Receptors: Role in Normal Physiology and Cancer

The individual contributions of ERα and ERβ in development and physiology have been elucidated with the use of ERα^−/−^, ERβ^−/−^, and double knockout mice (14). These mice survived to adulthood with a variable degree of phenotypic abnormalities in different tissues, including ovaries, uterus, mammary gland, prostate, testis, bone, and brain. These phenotypic alterations are ER type-specific, and they do not occur in the early phases of organ development. Both ERs are necessary for the development and proper function of the ovaries. ERα is involved in the development of the uterus and the mammary gland, as well as in bone physiology and male fertility, whereas ERβ is important for neuronal development (particularly in the somatosensory cortex) (4). Estrogens have a physiological role in the regulation of ion channels in epithelia of kidney, intestine, and lung to regulate whole body fluid and electrolyte balance (15). Estrogen has a physiological role as a regulator of Cl^−^ secretion in the colon, contributing to the salt and water retention observed during the high estrogen states (16, 17).

Consistent with their role in normal physiology, estrogens and ERs have been implicated in numerous human diseases including osteoporosis, obesity, cardiovascular and neurodegenerative diseases, and immune system disorders (2, 4, 18). Furthermore, and not surprisingly given the spectrum of their downstream signaling targets, estrogens contribute to cancer development and progression in different tissue types, mainly in endocrine-related reproductive tissues [mammary gland (19), ovary (20), uterus (21), and prostate (22)] and also in non-endocrine-related tissues [lung (23) and colon (24)]. The influence of ERα-mediated proliferative signaling has been extensively studied in the context of breast cancer, where pharmaceutical strategies to inhibit such signaling have been a clinical success in the past 20 years (19, 25). Inhibition of estrogenic signaling is achieved either through the use of selective estrogen receptor modulators (SERM) like tamoxifen and raloxifene, or through inhibition of estrogen synthesis via aromatase inhibitors. Proliferative signaling originating from ERα is also involved in the pathophysiology of non-reproductive tissues. For example, in the lung, ERα increases cellular proliferation of normal lung fibroblasts and lung tumor cell lines *in vitro* and tumor growth in immunocompromised mice (26), and is a negative prognostic marker in non-small-cell lung cancer independently of gender (27). While ERα promotes proliferation of prostate cancer cells, estrogen action through ERβ is anti-proliferative and anti-inflammatory in the prostate (28). ERβ has anti-proliferative effects in breast cancer when co-expressed with ERα, and studies show that loss of ERβ expression through promoter hypermethylation occurs frequently in ductal breast cancer (29). ERβ has also putative anti-proliferative effects in the ovary and endometrium (30, 31). Beside this, the role of ERβ in non-reproductive tissues has been studied particularly in the context of gastrointestinal malignancies.

## Evidence for Estrogen Receptor Signaling in Colon Cancer

ERβ is the predominant estrogen receptor expressed in both normal and malignant colonic epithelium, with limited or no expression of ERα observed in the colon (32). The expression of ERβ is reduced during colonic tumorigenesis as compared to normal tissue (12, 33, 34). ERβ is associated with stage and grade of disease, and an inverse relationship between ERβ expression and tumor progression has been reported in cell lines and clinical samples (35–37). It is therefore hypothesized that estrogen-mediated signaling exerts a protective role in CRC, further understanding of this may benefit cancer prevention and also provide additional therapeutic options for ERβ-positive tumors. The evidence for this anti-tumorigenic effect comes from a plethora of different studies, which are reviewed below.

### Epidemiological evidence for the role of steroid hormones in CRC

The incidence of CRC is higher in men than it is in women (38). With the exception of New Zealand and the USA (where rates are decreasing for both sexes), incidence rates are increasing in men while remaining steady among women (39, 40). Although not originally designed to investigate CRC risk, the Women Health Initiative study demonstrated that use of hormone replacement therapy (HRT) in post-menopausal women was associated with a 30% decreased incidence of CRC (41, 42). A number of other observational studies have addressed this question [summarized in Ref. (43)] and the majority report a decreased incidence of CRC among HRT users (although some studies do not report any difference), and a 30–60% decrease in CRC-related mortality. Interestingly, a recent study of 503 post-menopausal women has highlighted a role for ERβ in mediating the protective effects of HRT. Decreased CRC risk was associated with duration of HRT use specifically in ERβ-positive patients (OR for each 5 years interval: 0.87, 95% CI: 0.77–0.99) but not in ERβ-negative patients (OR: 1.02, 95% CI: 0.91–1.15) (44). This is an example of a relatively novel area of epidemiologic research that has been termed *molecular pathological epidemiology* (45). This type of study associates an etiological factor (like an exposure factor) to specific molecular changes in tumors, and analyzes interactive effects with the aim of gaining insights into the carcinogenic process. A number of these studies have analyzed in detail the exposure of post-menopausal women to hormone therapy, and have shown that this is associated with reduced risk of specific sub-sets of CRC, according to the expression of cell cycle regulators, DNA methylation, somatic mutations (BRAF and KRAS), and microsatellite instability (MSI) (46–49).

Incidence of CRC varies greatly by geographical region, with the highest rates seen in developed countries (Australia, New Zealand, Western Europe, and North America) and lowest rates in developing countries, particularly in Africa and Asia (38). These differences reflect distribution patterns for known risk factors for CRC (like smoking, obesity, and consumption of red meat), including differences in diet, e.g., Asian countries extensively use soy products containing phytoestrogens. In a recent meta-analysis of retrospective studies, soy consumption was found to be associated with a 21% decreased risk of CRC in women but not in men (50), a finding that might be related to the fact that soy supplementation is more effective when combined with estrogens *in vivo* (51). Furthermore, when people migrate from an Asian country to the U.S., incidence of CRC in the migrants begins to rise to the average of the U.S. (52).

Among the characteristics that are associated with the so-called *western* lifestyle, obesity is an important established risk factor for CRC in both men and women, although reported hazard ratios vary greatly according to whether body mass index (BMI) or waist circumference (WC) is used as a surrogate measure (53, 54). The prevalence of adenoma and advanced polyps is higher in men than women in all age groups except for people older than 70 years, and a positive association between BMI and the prevalence of colonic adenoma and advanced polyps was shown in young individuals of both gender and in premenopausal women according to hormonal status (53). This further supports the role that steroid hormones play in linking obesity to the increased risk of CRC, which has been previously demonstrated in animal models (55). Adipose tissue contributes to extra-gonadal testosterone aromatization and estrogen production. Adipose tissue is also an estrogen target tissue, and adipocytes express both ERα and ERβ, where they are involved in estrogen-mediated control of adipogenesis, lipogenesis, lipolysis and adipocyte proliferation (18). Obesity results in metabolic changes that can in turn promote colorectal carcinogenesis, and steroid hormones also modulate some of these mechanisms. For example, hyperinsulinemia and insulin resistance are associated with obesity, and insulin/insulin-like growth factor (IGF-I) signaling promotes tumorigenesis (56). Mitogenic leptin is also increased in obese patients (53, 57), and estrogens can regulate leptin through ERα (58). A decrease in anti-inflammatory adipokines is also linked to both obesity and risk of CRC, although in a cohort study of post-menopausal women this did not reach statistical significance in multivariate analysis (57). Obesity overall is characterized by low-grade chronic inflammation and reduced immunity, which also contribute to tumorigenesis (53). Furthermore, epidemiological studies have also linked physical activity to the risk of different cancers including CRC, demonstrating that increased exercise leads to reduced estrogen levels in post-menopausal women and a modulation of menstrual function in premenopausal women (59). Physical activity improves insulin resistance, reduces hyperinsulinemia, and reduces risk for diabetes; it might also reduce systemic inflammation alone or in combination with reduction in body weight through reducing inflammatory cytokines in adipose tissue (59).

### Clinical evidence for the role of ERβ in CRC progression

The expression of ERβ declines during tumor progression (34, 60), and analysis of clinical samples provided initial evidence that the reduced expression of ERβ could relate to tumor stage and grade, and to other characteristics of poor prognosis (including poorly differentiated tissue, vascular invasion, and decreased apoptotic index) (32, 33, 36). In a recent analysis of 1262 patients, expression of ERβ by immunohistochemistry correlated with prognosis in CRC: decreased expression was found in larger tumors of higher grade, and expression levels were also inversely correlated with Dukes stage. ERβ negativity was associated with a 54% increased risk of CRC-specific death, and a poorer disease-free survival (DFS) (35). Single-nucleotide polymorphisms (SNPs) located in the 5′ regulatory region of the ERβ gene (but not of ERα, progesterone receptor, or androgen receptor) were associated with improved survival after a diagnosis of CRC. These SNPs could be markers of receptor isoforms expression or CpG island methylation and transcriptional inactivation of ERβ (61). Furthermore, members of p160 ER co-activator superfamily AIB1 (SRC-3/NCoA3) and TIF2 (SRC-2), which interact with ERβ to mediate transcriptional activity, were up-regulated in adenomas and carcinomas compared to normal tissue; AIB1 was also associated with increased overall survival in a cohort of 110 CRC patients (62).

### Pre-clinical evidence of the role of ERβ in CRC

A number of *in vitro* studies supporting an inhibitory role for ERβ in CRC progression have been previously described (3). Recently, reported studies in mice have provided some *in vivo* evidence for the role of ERβ in CRC. Analysis of colonic tissue in ERβ^−/−^ mice showed that loss of ERβ leads to hyper-proliferation, de-differentiation, decreased apoptosis, and disruption of epithelial tight junctions; surprisingly no tumor formation was reported as hyper-proliferative cells moved faster to the surface of colonic crypts and were subsequently shed in the lumen (63). Saleiro and colleagues used the azoxymethane/dextran sodium sulfate-induced mouse model of colitis-associated CRC to show increased severity of clinical colitis in the absence of ERβ. This was coupled to increased inflammation and higher grade of dysplasia specifically in the early phase of tumor development, suggesting that ERβ might delay inflammation and neoplastic transformation (64). Giroux et al. crossed ERβ^−/−^ mice with Apc^Min/+^ C57BL/6J mice to obtain ERβ^−/−^ Apc^Min/+^ mice, which showed increased number and size of polyps, and tumors that were significantly larger than those in control Apc^Min/+^ mice. (65). These data suggest that loss of ERβ alone might not be sufficient to promote colorectal carcinogenesis, but that instead it would confer selective advantage to transforming epithelial colonocytes by eliminating a protective mechanism against genotoxic stress in the early phases of cellular transformation, therefore accelerating tumorigenesis.

As there are numerous downstream signaling pathways modulated by ERβ, there are a number of potential mechanisms by which ERβ exerts this protective effect. Gene expression profiles of ERβ^−/−^ Apc^Min/+^ mice suggested that modulation of the TGF-β signaling pathway contributed to the protective role of estrogens on intestinal tumorigenesis (65). Ectopic expression of ERβ in SW480 CRC cells resulted in inhibition of proliferation and cell cycle arrest in G_1_ phase, which was dependent on decreased *c-Myc* and altered expression of different cell cycle proteins. Furthermore, when ERβ-transfected SW480 cells were implanted in the fat pad of SCID/Beige mice in the presence of estrogen, ERβ over-expression resulted in a 65% reduction in tumor weight (37). Similarly, when ERβ-overexpressing HCT-116 cells were xenografted into BALB/c-nu nude mice, there was an 18% reduction in tumor growth, with a greater tumor reduction (89%) seen in combination with raloxifene (66). Apc^Min/+^ mice treated with a SERM (raloxifene) or an anti-estrogenic compound (Gonadorelin) showed a 65–75% inhibition in intestinal tumor multiplicity and size, and a 94–98% inhibition of polyps >2 mm in size, with decreased expression of β-catenin, cyclin D1, laminin 1b, and stem-like cells markers (67). Using the azoxymethane/dextran sodium sulfate model, Armstrong and colleagues showed that estrogen treatment reduced expression of ERβ while increasing ERα (68), and a similar finding was previously reported in rats treated with soy isoflavonoids (69), suggesting that the ratio of ERβ/ERα might have clinical implications in protecting against CRC (70). However, differential expression of ERβ isoforms in different tissues and organs, or during tumor progression, may also contribute to the outcome of estrogen signaling.

## ERβ Isoforms and Their Role in Colorectal Cancer

Four alternative isoforms of ERβ have been described so far (ERβ2/ERβcx, ERβ3, ERβ4, ERβ5), arising from alternative splicing of the last coding exon of the receptor; these proteins have a truncated or otherwise altered C-terminal domain (12). These isoforms were initially cloned from a human testis cDNA library and shown to have different pattern of tissue distribution (71). The C-terminal region of the ER contains the ligand-binding domain and is also involved in transcriptional activation, receptor dimerization, nuclear translocation and interaction with transcription co-regulators (4). Therefore, modifications in this particular region can have a profound impact on the activity and biological function of ERβ. A recent study addressed the functional role of ERβ isoforms, showing that ERβ2–4–5 are lacking intrinsic ligand-dependent transactivation activity, making ERβ1 (wild-type) the only functional receptor isoform. However, ERβ2–4–5 form heterodimers with ERβ1, and they can enhance transcriptional activity induced by ERβ1 at physiological concentrations of estrogen (72). ERβ isoforms were identified in two CRC cell lines (HCT8 and HCT-116) by RT-PCR, with two more cell lines (DLD-1 and LoVo) reported negative for all isoforms (73). ERβ2 and ERβ5 were the predominant isoforms identified in 91 primary colorectal carcinoma samples by immunohistochemistry, using custom-made antibodies raised against isoform-specific peptides in the C-terminal region (74). ERβ2 was decreased in tumor tissue compared to normal adjacent tissue, and associated with right-sided tumors and the presence of lymph node metastases, while ERβ5 was expressed in all tumor samples and also in a panel of 20 CRC cell lines (74). The specific role of ERβ isoforms in CRC is yet to be fully elucidated; however, it is plausible that the co-expression of different isoforms of the receptor (namely ERβ2 and ERβ5), even in the presence of low levels of full-length ERβ, would complicate the estrogen-mediated signaling involved in tumor suppression, and therefore, this needs to be taken into account when designing therapeutic strategies targeting the estrogen pathway.

## Estrogen Receptors, Tumor Location, and Microsatellite Instability

Colorectal cancers can arise from different regions of the colon, and their location impacts on their biological characteristics. Specifically, the right (proximal) colon and the left (distal) colon have different embryological origins (the midgut and the hindgut, respectively), which are reflected in differences in histopathology, molecular biological patterns, metastatic spread, and mutational profile of tumors (75–77). Proximal and distal tumors also have differences in their respective microbial flora, which can impact on tumor progression (78). Proximal tumors have a higher frequency of MSI (79), a pathway associated with loss of mismatch repair proteins resulting in accumulation of errors in DNA replication. Estrogens appear to be somehow linked to the left-right dichotomy in CRC: women develop more proximal, and men more distal colon and rectal cancers, and female sex is an independent predictor of proximal cancers (80). Furthermore, the incidence of proximal cancers in women increases with age, while the same is not observed in men (80). The reduction of ERβ expression is more prominent in proximal CRC (21%) than in distal cases (7%) (36), providing a putative rationale for the aforementioned gender disparities. MSI occurs more frequently on the right side of the colon, and in female patients (81), and a recent analysis reports an association of MSI with proximal cancers, hypermethylation (CIMP phenotype), and inflammatory subtype of CRC (79). Endocrine factors are associated with MSI tumors (82), with a significant interaction among age, sex, and MSI: women are more likely than men to have MSI tumors at an older age (82). This can be explained, at least in part, by the ability of estrogen to induce the expression of mismatch repair proteins (MLH1 and MSH2) via ERβ (83).

Epidemiological differences have also been reported according to tumor location. Early ulcerative dysplastic lesions in the left colon are more likely to progress than those originating in the right colon (84). However, mortality rates are higher in right colon than in left-colon cancers (75, 79). Molecular pathological epidemiology studies have also provided some evidence that post-menopausal hormone therapy use could be associated with reduced risk of CRC selectively for distal tumors that are KRAS wild-type and MSI-low (48, 49).

The division between left and right colon might not be as dichotomous as previously thought, and instead represent a more homogenous continuum along the colon length (85). This is supported by linear changes in the distribution pattern of different molecular markers along the intestine (86, 87), and also by changes in recurrence rates according to tumor location (88). Furthermore, a recent study showed that within the right colon, there are differences between more distal and more proximal regions, where higher percentages of stage IV, node-positive or metastatic cancers, and higher mortality rates are reported in the more proximal portion (transverse colon) of the right colon (89).

Although more studies are needed to address the role of estrogens and differences in ERβ expression in different colon locations, the data reviewed here suggest that estrogen signaling might be (at least in part) responsible for the gender gap in proximal versus distal colon tumors and associated differences in biology and mortality rates.

## Estrogen-Mediated Regulation of Tumor Microenvironment and Immune Surveillance

Estrogen and other ER ligands modulate both innate and adaptive immunity, which is reflected in gender differences during autoimmunity (90). The contribution of inflammatory networks and immune-escape mechanisms in the tumor microenvironment in colorectal carcinogenesis is well established (91, 92). Yet, how ER-dependent pathways contribute to the regulation of the inflammation within the tumor microenvironment is not well understood. Analysis of genome-wide gene expression changes in three CRC cell lines transfected with ERβ showed that IL-6 signaling pathway represented a significantly enriched sub-network, and RT-PCR confirmed the down-regulation of IL-6 (as well as 10 different IL-6 target genes) by ERβ in SW480 cells (93). This has important potential consequences in modulating a tumor-promoting microenvironment, as IL-6 activate the STAT3 transcription factor in epithelial, myeloid cells, and myofibroblasts, promoting CRC tumorigenesis (94, 95). Different ER ligands regulate the homeostasis of bone marrow myeloid and lymphoid progenitors of dendritic cells (DC), and also modulate DC activation and production of inflammatory mediators (96). Estrogen signaling promotes the granulocyte macrophage-colony-stimulating factor (GM-CSF)-mediated differentiation of myeloid progenitor cells into CD11b^+^ DC, through ERα-mediated activation of interferon regulatory factor 4 (IRF4) (97). Estrogen affects DC to regulate response to TLR agonists (98). Therefore another plausible mechanism by which estrogen could exert anti-tumorigenic effects in CRC would be through the regulation of DC-mediated immune surveillance, although this hypothesis needs to be addressed experimentally. Furthermore, the estrogen signaling in immune cells is complicated by multiple effects on different cell types, as estrogen is also known to have anti-inflammatory effects on monocytes and macrophages, and to enhance CD4^+^ T cell response (96). ERβ is also involved in mediating anti-inflammatory responses. Using human osteosarcoma, U2OS cells expressing ERβ as a model, Cvoro and colleagues showed that both estradiol and ERβ-selective agonists reduced the activation of 18 pro-inflammatory genes (including TNF-α, IL-6, and CSF2) induced by TNF-α (99). This suggests that selective activation of ERβ-mediated signaling might have a negative impact on tumorigenesis also through down-regulation of pro-tumorigenic inflammatory signaling in the tumor microenvironment. Moreover, cytokines produced by the tumor microenvironment could feedback on estrogen signaling through increased 3β-hydroxysteroid dehydrogenase gene expression, therefore inducing the biosynthesis of active steroid hormones from the inactive adrenal steroid dehydroepiandrosterone in peripheral target tissue (100). Finally, work with the ERβ^−/−^ mouse model showed that a diet rich in estrogenic isoflavones and fiber could modify the composition of intestinal microbiota through ERβ in female mice, a finding, which have important consequences since the microbiota is known to influence local inflammation and intestinal tumorigenesis (101).

## ER Selective Ligands and Potential Avenues for Targeted Therapy

Plant-derived flavonoids bind to both ERα and ERβ and are considered protective agents against endocrine-dependent tumors, among other potentially beneficial effects on human health. This provides a mechanistic explanation for some epidemiological evidence linking diet to CRC risk. In the specific context of colorectal carcinogenesis, naringenin (5,7,4′-trihydroxyflavone) and quercetin (2-3,4-dihydroxyphenyl-3,5,7-trihydroxy-4H-1-benzopyran-4-one) induced apoptosis in CRC cells (102, 103). Both compounds activate the p38/MAPK signaling leading to activation of pro-apoptotic caspase 3 in DLD-1 colon cancer cells expressing ERβ. Quercetin also increased the expression of the oncosuppressive protein PTEN and decreased cyclin D1 promoter activity (103). However, the potential medical use of flavonoids in cancer has not been addressed beyond pre-clinical studies.

Other chemical compounds used in industry and agriculture (termed xenoestrogens, or endocrine-disrupting chemicals) negatively impact on human health and have colorectal-specific effects. For example, bisphenol A (BPA) bind to both ERα and ERβ and act as an estrogen antagonist by inhibiting estrogen-induced pro-apoptotic signaling and gene expression (104). An increased research-driven awareness of the harmful effects of endocrine disruptors in recent years have resulted in policy changes concerning the use of these compounds in human activities, although the broad impact of this policy implementation on human disease is yet to be fully elucidated, and more basic research is needed to address mechanisms of action and physiological roles of xenoestrogens (105).

The development of ER subtype-selective ligands has been a very active field of research over the past 10 years. Consequently, a large number of ERβ-specific agonists have been designed, although very few show potential as anti-cancer drug candidates (106). Isoquinolinone derivatives have been tested on different cancer cell lines and inhibit C-26 CRC cell proliferation. SR16388, another compound with ERβ-specific activity, has shown promising properties for the treatment of non-small cell lung cancer, CRC, and androgen-independent prostate cancer (106).

## Conclusion

**Figure 1:**
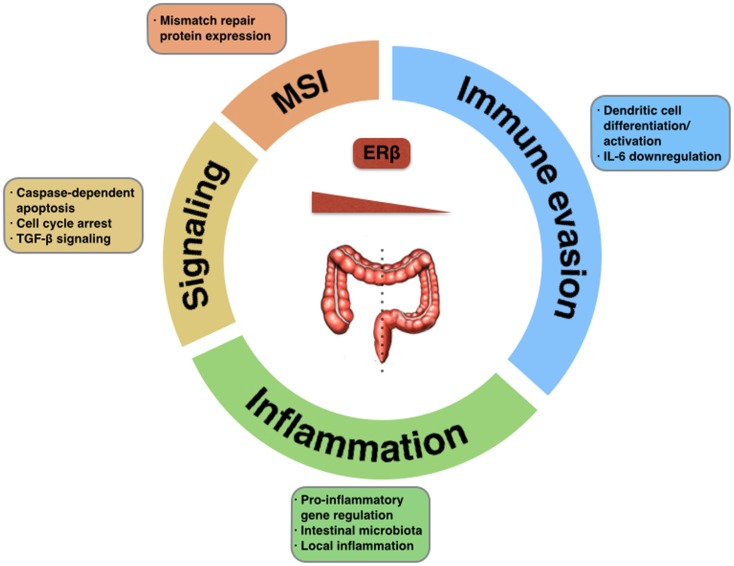
**Tumor-suppressive functions of ERβ in CRC**. The potential impact of estrogen signaling through ERβ in colorectal carcinogenesis could be exerted via activation of pro-apoptotic signaling, regulation of mismatch repair proteins, modulation of the inflammatory tumor microenvironment, activation of immune surveillance mechanisms, or down-regulation of immune evasion mechanisms. Selective loss of ERβ in different location within the large intestine promotes tumorigenesis.

Estrogen signaling has an anti-tumorigenic role in the colonic mucosa, through selective activation of pro-apoptotic signaling mediated by ERβ, inhibition of inflammatory signals, and modulation of the tumor microenvironment and different immune surveillance mechanisms (Figure 1). Selective loss of ERβ expression during CRC progression highlights the importance of this oncosuppressive signaling, and provides difficulties in using this same signaling as a therapeutic target.

Two main challenges characterize the future of this research field. First, it is of crucial importance to further characterize ERβ expression in patients, identifying a specific subset of patients with decreased ERβ loss or differential expression of ERβ isoforms, in order to help stratification and patient selection for future therapies targeting estrogen signaling. Some of these questions are currently being investigated (107). Different therapeutic strategies are being studied to selectively target ERβ-dependent signaling, requiring the concomitant development of an appropriate diagnostic companion tool. Second, the down-regulation of ERβ and the subsequent loss of protective signaling in advanced tumors warrant increased research into the mechanisms involved, to address the potential for therapeutic re-activation of oncosuppressive signaling. A positive feedback mechanism has been described *in vitro* where estrogen stimulation leads to increased ERβ protein expression, reinforcing pro-apoptotic signaling in colon cancer cells (108). This mechanism is lost during colon cancer progression due to unknown factors. Studies in breast cancer, where ERβ-selective loss is also reported, suggest that epigenetic mechanisms (namely, promoter methylation and histone acetylation) might be involved. Treatment of breast cancer cells with a combination of de-methylating agents and histone deacetylase (HDAC) inhibitors fully restored ERβ expression (109), and recently a therapeutic approach with combinations of HDAC inhibitors and SERMs (hybrid drugs termed SERMostats) is being investigated in breast cancer (110). In CRC, the adenovirus-mediated induced re-expression of ERβ showed promising results in pre-clinical studies (111). The design of novel and specific SERMs with selective differential anti-estrogen and estrogen-like activities in different target tissues will greatly advance clinical development of therapeutic strategies for the estrogen pathway. It will be crucial to overcome toxicity issues related to current SERMs and to develop strategies allowing to conserve beneficial estrogen effects in certain tissues while simultaneously targeting signaling involved in cancer initiation and progression. One such strategy has recently emerged, combining SERMs and estrogens in what has been named *tissue selective estrogen complex* (TSEC), to elicit a blend of ER agonistic activities (112).

## Conflict of Interest Statement

Francesco Caiazza is supported by a Newman Fellowship awarded by the UCD Foundation and funded by a donation from Merck Serono. The authors have no other relevant affiliations or financial involvement with any organization or entity with a financial interest in or financial conflict with the subject matter or materials discussed in the manuscript.
